# Regulation of Stem Cell Differentiation by Inorganic Nanomaterials: Recent Advances in Regenerative Medicine

**DOI:** 10.3389/fbioe.2021.721581

**Published:** 2021-09-30

**Authors:** Fumei He, Jinxiu Cao, Junyang Qi, Zeqi Liu, Gan Liu, Wenbin Deng

**Affiliations:** School of Pharmaceutical Sciences (Shenzhen), Sun Yat-sen University, Shenzhen, China

**Keywords:** Inorganic nanomaterials, stem cells, differentiation, cell therapy, regenerative medicine

## Abstract

Transplanting stem cells with the abilities of self-renewal and differentiation is one of the most effective ways to treat many diseases. In order to optimize the therapeutic effect of stem cell transplantation, it is necessary to intervene in stem cell differentiation. Inorganic nanomaterials (NMs), due to their unique physical and chemical properties, can affect the adhesion, migration, proliferation and differentiation of stem cells. In addition, inorganic NMs have huge specific surface area and modifiability that can be used as vectors to transport plasmids, proteins or small molecules to further interfere with the fate of stem cells. In this mini review, we summarized the recent advances of common inorganic NMs in regulating stem cells differentiation, and the effects of the stiffness, size and shape of inorganic NMs on stem cell behavior were discussed. In addition, we further analyzed the existing obstacles and corresponding perspectives of the application of inorganic NMs in the field of stem cells.

## Introduction

Stem cells refer to cells with self-renewal and differentiation capacity, which can be roughly divided into embryonic stem cells and somatic stem cells. Embryonic stem cells are derived from blastocysts (Reubinoff et al., 2000). Typical somatic stem cells include mesenchymal stem cells (MSCs), neural stem cells (NSCs), hematopoietic stem cells (HSCs) and so on. Terminally differentiated somatic cells can be reprogrammed into induced pluripotent stem cells (iPSC) with embryonic stem cells (ESCs)-like properties by transfection of defined factors (Takahashi and Yamanaka, 2006), and further differentiate into different cell types. With the increasing of research on stem cells in recent years, more and more evidence is emerging that stem cell transplantation is one of the most effective methods to treat neurological diseases, bone injury and other diseases (Yang et al., 2018; Krukiewicz et al., 2020). The growth and differentiation of stem cells are easily affected by their surrounding matrix. Changing the size, hydrophilicity, roughness, and arrangement of the cell attachment surface can directly affect cell behavior (Zanden et al., 2014). To be able to fully realize the therapeutic potential of stem cells in the field of regenerative medicine, precise control of the fate of stem cells is one of the first issues to be addressed (Solanki et al., 2013b).

Inorganic NMs have been widely used in bioimaging, drug delivery, biosensing, photothermal therapy, and 3D printing due to their own excellent properties (Wu et al., 2018; Mashayekhi et al., 2020; Tavares et al., 2021). In recent years, inorganic NMs have been widely used to manipulate the fate of stem cells. Inorganic NMs exert their influence on stem cell behavior as unique biomolecules, besides that, as modifiable non-viral transfection vectors, inorganic NMs can carry various bioactive molecules that regulate stem cell behavior, including RNA, plasmids, proteins, or polypeptides, etc., thereby further stimulating the proliferation, migration, differentiation and paracrine behavior of stem cells. Various inorganic NMs, including graphene (Rostami et al., 2020), carbon dots (Shao et al., 2017), gold nanoparticles (AuNPs) (Heo et al., 2014), silver nanoparticles (AgNPs) (Wan et al., 2020), nano titanium-based alloys (Jalali et al., 2020), strontium nanoparticles (Hu et al., 2017), iron oxides nanoparticles (Zhang et al., 2020), manganese dioxide (MnO_2_) nanoparticles (Wang et al., 2017), silicon dioxide (SiO_2_) nanoparticles (Gandhimathi et al., 2019), and black phosphorus (BP) nanosheets (Xu et al., 2020), have been extensively explored in stem cells regeneration medicine. The stiffness, size and shape of inorganic NMs can directly affect the bioactivity of materials and, in turn, affect the differentiation of stem cells (Huang et al., 2020). In this paper, we summarized the progress of various inorganic NMs on the regulation of stem cells differentiation, and the physical properties of inorganic NMs in regulating stem cell differentiation were discussed. In addition, the obstacles to the application of inorganic NMs and the corresponding solutions were further analyzed.

### Carbon-Based Nanomaterials

Carbon-based materials such as carbon nanotubes (CNTs) and graphene have good physical properties, stability and biocompatibility, which can maintain the adhesion and proliferation of stem cells and can influence the differentiation fate of stem cells (Lee et al., 2011; Shao et al., 2018; Gupta et al., 2019). But different types of carbon-based materials show different effects on stem cells. Compared with graphene, iPSCs are more likely to grow on the surface of graphene oxide (GO) because the oxygen-containing functional groups of GO greatly improve the surface hydrophilicity, which is conducive to cell adhesion, growth and differentiation (Liu et al., 2011; Feng et al., 2018; Yang et al., 2018) compared the effects of CNTs, GO, and graphene on dopamine neural differentiation of mESCs. Only GO was found to significantly enhance the differentiation of ESCs into dopaminergic neurons (Yang et al., 2014a). GO can improve biological activity during biomineralization and promote osteoblast adhesion (Krukiewicz et al., 2020). Go can also promote the attachment and proliferation of MSCs, which promotes spontaneous and stimulated osteogenic differentiation (Rostami et al., 2020). In addition, carbonaceous nanomaterials called fullerenes are a potential material for inducing osteogenic differentiation of MSCs (Yang et al., 2014b). Gadofullerene nanoparticles effectively reduce reactive oxygen species (ROS) levels in blood and promote erythrocyte maturation (Jia et al., 2020).

Being able to simultaneously monitor and direct stem cell differentiation is important for the application of stem cells. Kim et al. (2013a) reported a non-invasive and rapid electrochemical method to detect the differentiated state of NSCs using 3D GO encapsulated AuNPs based on the feature that there are many C=C unsaturated groups on the surface of NSCs, while the unsaturated groups significantly decrease after differentiation. Shao et al. (2017) used citric acid-based carbon dots (CDs) to label rat bone marrow mesenchymal stem cells (BMSCs) without affecting cell viability to provide real-time monitoring of cell activities. Meanwhile, the presence of CDs could enhance the osteogenic differentiation efficiency of rBMSCs by promoting matrix mineralization and up-regulating the expression of osteoblast gene markers alkaline phosphate (ALP), runt related transcription factor 2, osteocalcin and bone sialoprotein. Similarly, (Meng et al., 2019; Yang et al., 2019), synthesized Mg^2+^-doped CDs and Zn^2+^-doped CDs, which were internalized by cells as a biomarker and simultaneously promoted the osteogenic differentiation of mouse embryo osteoblast precursor cells (MC3T3-E1) by increasing their ALP activity.

### Metal-Based Nanomaterials

#### Gold Nanoparticles

AuNPs possess good biocompatibility and can serve as an ideal alternative material to promote bone tissue regeneration. AuNPs significantly promote osteogenic differentiation and mineral deposition in MSCs (Suarasan et al., 2015; Mahmoud et al., 2020). *In vivo* experiments have shown that AuNPs can promote bone regeneration at bone defect sites and play a positive role in bone healing (Heo et al., 2014).

AuNPs as vehicles also play an important role in the applications of stem cells. AuNPs deliver basic fibroblast growth factor and bone morphogenetic protein-2 (BMP2) to promote osteogenic differentiation of MSCs (Qi et al., 2017). Patel et al. (2014) constructed a mimic transcription factor NanoScript based on AuNPs, which could achieve nuclear localization and initiate the transcriptional activity of both reporter plasmids and endogenous genes, and successfully initiate selective differentiation of adipose derived mesenchymal stem cells (ADMSCs) into myoblasts (Patel et al., 2015c). Next, they designed NanoScript to depress the expression of SOX9 gene in NSCs and promote the formation of functional neurons (Patel et al., 2015a). After that, they modified the NanoScript which specifically enhanced SOX9 gene expression with a small molecule to enhance the chondrogenic differentiation of ADMSCs by increasing the activity of histone acetyltransferases (Patel et al., 2015b). Wu et al. (2020) developed multifunctional AuNPs to control and detect osteogenic differentiation of hMSCs in real time, giving AuNPs multiple applications in stem cell regenerative medicine.

#### Silver Nanoparticles

AgNPs have promising anti-inflammatory and antimicrobial activities (Hebeish et al., 2014; Xia et al., 2020). Topical coating facilitates the healing of wounds (Tian et al., 2007). Implanting stem cells into dressings containing AgNPs also promotes cell growth and wound healing (Gao et al., 2020). Silver nanomultilayers decorated on the surface of titanium alloy implants enhance osteogenic differentiation of rBMSCs (Wan et al., 2020). Therefore, AgNPs which possess both antibacterial and osteogenic differentiation abilities are potential biomaterials for treating infectious bone defects (Li et al., 2020a). AgNPs induce osteogenic differentiation independent of Ag^+^ (Qin et al., 2014), but associated with the increase of intracellular ROS (Chowdhury et al., 2018; Dayem et al., 2018).

#### Nano Titanium Alloys

Titanium (Ti)-based alloys are a common type of bone implants. Inert Ti alloy implants can quickly form TiO_2_ film on the surface *in vivo*, and the film can be recovered in a short time after disruption. Therefore, Ti alloy implants have good biocompatibility (Zhang and Chen, 2019).TiO_2_ films enhance the osseointegrative properties of the orthopedic implant material (Blendinger et al., 2021). Ti-based materials such as Ti-modified TiO_2_ nanotubes can promote the adhesion, proliferation and osteogenic differentiation of MSCs, and *in vivo* transplantation can improve osteoporosis (Yu et al., 2017). At the same time, they promote the adhesion of macrophages and the transformation of M1-to-M2, which induces a favorable immune microenvironment for bone fusion (Yang et al., 2020). TiO_2_ nanotubes can promote F-actin polymerization and osteogenic differentiation in BMSCs (Liu et al., 2021). Apart from that, TiO_2_ nanotubes can target drugs to bone and enhance osteoblast differentiation (Hashemi et al., 2020; Tong et al., 2020).

#### Strontium Nanoparticles

Strontium (Sr) has biological effects to promote osteogenesis, and moderate supplementation of Sr enhances calcium absorption (Nielsen, 2004). Zhang et al. (2013) loaded Sr on TiO_2_ nanotubes to obtain Crystalline SrTiO_3_, which can realize the slow release of Sr. It further enhances the ALP activity and matrix mineralization ability of MSCs. SrTiO_3_ nanotube arrays have good biocompatibility and are ideal implants for osteoporotic bone (Xin et al., 2009). Meanwhile Sr can depress the activity of osteoclasts, which greatly improves the osteogenesis (Hu et al., 2017). In addition, Sr also promotes M2 type polarization of macrophages and reduces proinflammatory factor to create a favorable environment for bone healing (Li et al., 2018).

#### Iron and its Oxides-Based Nanoparticles

Fe_3_O_4_ nanoparticles (Fe_3_O_4_ NPs) are typical magnetic materials for promoting bone tissue regeneration (Zhang et al., 2020). Fe_3_O_4_ NPs composite scaffolds could enhance adhesion, proliferation and osteoconduction of hMSCs (Bock et al., 2010; Kim et al., 2020b). Magnetite-modified scaffolds facilitate the adhesion and proliferation of cells, which in turn promotes osteogenic differentiation of MSCs and osteogenesis *in vivo* (Lee et al., 2019b; Pistone et al., 2019; Xia et al., 2019). (Li et al., 2020c) treated hMSCs with Fe_3_O_4_ NPs and obtained exosomes labeled with Fe_3_O_4_ NPs, which significantly promoted proliferation, migration, and angiogenesis of human umbilical vein endothelial cells (hUVECs) in the skin injury model.

#### Manganese Dioxide-Based Nanoparticles

One of the most important applications of MnO_2_ in stem cell regenerative medicine is bio-imaging, in which MRI is used to track the distribution of stem cells *in vivo* (Yang et al., 2018). MnO_2_ nanotubes are reduced to Mn^2+^ in acidic solution or by intracellular glutathione and further activate magnetic resonance imaging (MRI) (Lu et al., 2017; Wu et al., 2018). Mn^2+^ can promote neural differentiation and neurite growth of rat pheochromocytoma cells (PC12). Moreover, the *ππ* stacking interaction affects the charge-carrier density between catecholamines and MnO_2_ nanoellipsoids through which the catecholamines secreted by PC12 cells can be monitored in real time (Kim et al., 2013a).

MnO_2_ as an antioxidant can alleviate the oxidative environment of injured tissues. Modification of MnO_2_ on the surface of MSCs can improve cell survival in oxidative disease tissue while secreting more proangiogenic factors (Teo et al., 2019). MnO_2_-modified hydrogel significantly reduces the ROS at the site of spinal cord transection injury in rats and promotes the neural differentiation of the implanted MSCs (Li et al., 2019). Based on the large specific surface area and molecular characteristics, (Yang et al., 2018), used MnO_2_ 3D nanoscaffolds to deliver hNSCs and small molecules to the spinal cord injury site in mice, improving cell survival and facilitating repair. BMP2 loaded MnO_2_ nanoparticles can enhance the recruitment of skeletal stem cells to promote bone repair (Li et al., 2020b).

### Non-Metallic Nanomaterials

#### Silicon Dioxide Nanoparticles

Silicon is one of the essential mineral elements in human body, which plays an important role in the formation and maintenance of human bones. Soluble silicon dioxide plays a dual role in bone metabolism. On the one hand, it can promote osteoblasts. On the other hand, it can inhibit the formation of osteoclasts and bone resorption (Mladenovic et al., 2014). Therefore silicon holds great promise for osteoporosis therapy (Price et al., 2013). SiO_2_ can enhance the hydrophilicity of nanofibers and favor the adhesion and growth of MSCs, which in turn promote osteogenic differentiation (Gandhimathi et al., 2019).

Mesoporous silica nanoparticles (MSNs), with excellent adsorption properties, stability and biocompatibility, are often used as vehicles for various active molecules to direct the fate of stem cells (Mashayekhi et al., 2020). BMP2 delivery by MSNs can promote the differentiation of MSCs towards osteoblasts (Zhou et al., 2015). Solanki et al. (2013a) developed a SiO_2_ nanoparticle-mediated reverse uptake platform that delivered siRNA to depress SOX9 expression and enable NSCs to differentiate into neurons. Tavares et al. (2021) used functionalized MSNs as inorganic bone building blocks of multi-bioactive nanocomposite bio-ink, and used 3D bio-printing technology to generate biomaterials containing MSCs, which opened up great potential for bone tissue engineering to fabricate living 3D structures.

#### Black Phosphorus Nanomaterials

Black phosphorus, as a new type of semiconductor material, possesses good optical and electrical properties. In addition, BPNMs have many advantages such as large specific surface area, high photothermal conversion efficiency, good biocompatibility and biodegradability, which are widely used in biological fields (Yin et al., 2017). BPNMs are not only widely used in photothermal therapy of tumors (Luo et al., 2020), (Chen et al., 2018) also reported neuroprotective effects of BPNMs. BP hydrogel scaffolds combined with electrical stimulation can significantly promote the transformation of BMSCs into neuro-like cells (Xu et al., 2020). In addition, BPNMs can promote the proliferation, migration and osteogenic differentiation of stem cells and is widely used in bone repair (Lee et al., 2019a; Raucci et al., 2019). BP degradation produces phosphate ions. Hydrogels encapsulated with BPNMs can capture free calcium ions *in vivo* to form calcium salts, thus accelerating the biomineralization of bone defects and enhancing bone regeneration (Huang et al., 2019). BP hydrogel promotes osteogenic differentiation of hMSCs *in vitro* and shows the fastest rate of bone formation when transplanted into a rat model of skull defects (Miao et al., 2019). Pan et al. (2020) synthesized a chitosan thermosensitive hydrogel containing BPNMs for rheumatoid arthritis treatment. BP can eliminate the hyperplastic synovial tissue under the irradiation of near-infrared light, so as to relieve inflammation. Meanwhile, BPNMs continue to degrade *in situ* and release phosphate ions to realize the mineralization of calcium for bone regeneration.

### Physical Properties of Inorganic Nanomaterials Modulate Stem Cell Differentiation

#### Stiffness

The stiffness and elasticity of the extracellular matrix (ECM) determine the differentiation fate of stem cells (Trappmann et al., 2012). Modulating the stiffness of nanomaterials can direct the fate of stem cells. Neural crest stem cells differentiate into smooth muscle cells around stiff substrates and into glial cells in softer matrix (Zhu et al., 2019). Similarly, MSCs mainly differentiate toward neurons in the soft matrix similar to nerve tissue, toward myocytes in the medium hardness matrix, and toward osteoblasts in the high hardness matrix (Engler et al., 2006). Wang et al. (2020) enhanced the efficiency of osteogenic differentiation by improving the hardness of gradient nanostructured Ti materials.

#### Shape

The microstructure of the cellular matrix greatly influences the growth and differentiation of cells. Solanki et al. (2010) found that the arrangement of the ECM affects the differentiation of NSCs, which are more likely to differentiate into neurons on grid-shaped ECM than strip or square. Later, they proved that the nanotopographic cues of the carbon nanotube network could cooperatively induce the selective growth of hNSCs (Park et al., 2011). Nanostructure modification on the surface of titanium grafts can promote osseointegration (Souza et al., 2019). Zhao et al. (2010) found that titanium microstructure surface can promote osteogenesis-related gene expression in osteoblasts, but has a down-regulation trend in cell proliferation, total protein formation, ALP activity and cell matrix mineralization. However, the addition of nanostructures on the surface of titanium microstructures significantly promotes osteogenic differentiation. At the same time, there is no significant difference between the total protein content and ALP activity on the nanostructured surface and the smooth surface, but the micro/nano surface features significantly increase the expression of both. *In vivo* transplantation can also promote new bone formation and osseointegration at the femoral defect (Zhang et al., 2013; Li et al., 2016a; Yuan et al., 2018). These results indicate that the micro/nano structure has a synergistic effect on promoting bone regeneration.

#### Size

There have been many studies reporting the effect of the size of nanomaterials on stem cell behavior. Stem cells exhibit different adhesion, proliferation, migration and environmental stress responses on different sizes of nanostructured substrates (Kim et al., 2020a). The osteoinduction activity of AuNPs at 20 nm is higher than that at 40 nm (Li et al., 2016b). However, (Li et al., 2017), reported that AuNPs below 10 nm significantly decreased osteogenesis-related gene expression in BMSCs, but increased the expression of genes related to adipogenesis and the formation of oil droplets. Zhang et al. (2015) demonstrated that AgNPs with an average diameter of 10 ± 5 nm could promote the proliferation and osteogenic differentiation of mMSCs *in vitro*. However, AgNPs with a mean diameter of 43 ± 11 nm could promote adipogenic differentiation of hMSCs and inhibit osteogenic differentiation in the early stage of differentiation (He et al., 2016). So, the size of AgNPs has a significant effect on the differentiation of MSCs. Shen et al. (2015) demonstrated that Ti nanoparticles with large particles (80 nm) greatly promoted the proliferation and differentiation of MSCs compared with other small particles (20 and 40 nm). The ability of TiO_2_ to promote osteogenic differentiation of MSCs is enhanced with the increase of nanotube diameter in a certain range (74–148 nm) (Tong et al., 2020). In contrast, when compared with 100 nm, TiO_2_ nanotubes with a size of 15 nm were reported by (Park et al., 2009) to be the best for both mMSCs and HSCs to promote adhesion, proliferation and differentiation. These conflicting conclusions led us to be cautious when looking at the size issue, and further determine the optimal size in combination with the preparation process of nanomaterials and cell types. In addition, the size of nanomaterials is often closely related to biological toxicity, which should not be ignored.

**FIGURE 1:**
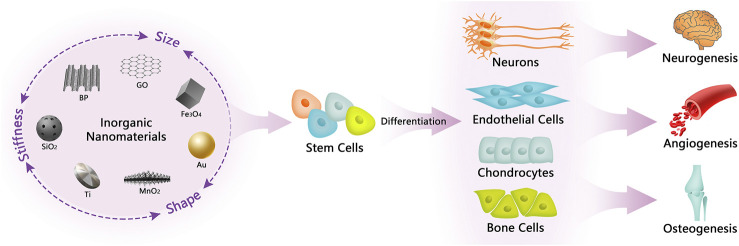
Illustration of inorganic NMs in regulating stem cell differentiation and their biological applications. The physical properties of inorganic NMs, including stiffness, shape, and size, can influence the guidance of inorganic NMs to the fate of stem cells, which in turn mediate the differentiation of stem cells to produce different kinds of functional cells that are beneficial for neurogenesis, angiogenesis, and osteogenesis.

Taken together, a variety of inorganic NMs are capable of intervening in the differentiation of stem cells and are used for the treatment of specific diseases. Meanwhile, the physicochemical characteristics including stiffness, shape and size of inorganic NMs greatly influence their differentiation guidance (see Figure 1).

### Obstacles and Solutions for the Application of Inorganic Nanomaterials

We searched the literatures through the PubMed website using “stem cells and nanomaterials” as keywords in the past 5 years. A total of 3,526 results were displayed, and only two remained after adjusting article type to clinical trial, both about nanocurcumin. In addition, our keywords search for “nanomaterials and nanoparticles” in the *ClinicalTrials.gov* database yielded only four results and none of the clinical studies were related to stem cells, so we can see the extremely low clinical translation of nanomaterials. Combined with the analysis of the current published papers on the effects of inorganic NMs on stem cell differentiation (see Table 1), it is not difficult to find that the research methods and contents are monotonous and repetitive. Most of the papers just simply characterize some differentiation markers *in vitro* and *in vivo*. Articles usually emphasize that inorganic NMs possess good biocompatibility. However, these experiments only carry out short-term tests *in vivo* and *in vitro*, and even do not detect the distribution of nanoparticles in various organs in the body, which is far from the actual clinical application. In addition, due to the profound differences in anatomy, physiology and genetics between humans and animals (Su et al., 2018), successful animal experiments will not necessarily be replicated in humans. The safety problem is one of the important reasons for the failure of clinical translation (Arrowsmith, 2011). In the absence of complete safety data, clinical failure of nanomaterials is inevitable. As for the research strategies of nanomedicine, (Su et al., 2018), put forward critical and balanced suggestions, including multi-field cooperation, encouraging research to focus more on the biosafety of nanoparticles rather than the efficacy, and establishing standardized evaluation methods.

**TABLE 1:** A summary of inorganic nanomaterials guiding stem cell differentiation and its application in regenerative medicine.

Types of nanomaterials	Cell sources	Animal model	Cell lineages generated/effectiveness	*In vitro*/*in vivo*	Underlying mechanism	References
GO/Poly(methyl methacrylate) composite scaffolds	hMSCs		Enhanced osteogenic differentiation	*In vitro*		Krukiewicz et al. (2020)
PCL/GO nanocomposite scaffolds	rBMSCs		Enhanced osteogenic differentiation	*In vitro*		Rostami et al. (2020)
CDs	rBMSCs		Enhanced osteogenic differentiation	*In vitro*	ROS-mediated MAPK pathway	Shao et al. (2017)
Mg^2+^-doped CDs, Zn^2+^-doped CDs	MC3T3-E1		Enhanced osteogenic differentiation	*In vitro*		Meng et al. (2019); Yang et al. (209)9
CNTs	mNSCs		Promoted neuronal differentiation and neurite outgrowth	*In vitro*	Integrin-mediated interactions between NSCs and CNT multilayers	Shao et al. (2018)
CNTs	mouse hippocampal neuronal cells (HT-22)		Enhanced neural cell adhesion and neurite outgrowth	*In vitro*		Gupta et al. (2019)
Graphene-based mat	rADSCs		Enhanced Neurogenic differentiation	*In vitro*		Feng et al. (2018)
AuNPs/gelatin hydrogels	hADSCs	rabbit Parietal bone defects	Enhanced osteogenic differentiation	*In vitro* and *in vivo*		Heo et al. (2014)
AuNPs	hMSCs		Enhanced osteogenic differentiation	*In vitro*	Silenced the adipogenic-related gene peroxisome proliferator-activated receptor *γ* (PPARγ)	Wu et al. (2020)
Core-Shell Mesoporous Silica Containing AgNPs	rBMSCs		Antibacterial activity and osteogenic differentiation	*In vitro*		Li et al. (2020a)
AuNPs	hMSCs		Promoted adipogenesis	*In vitro*	Induced cellular ROS level	Li et al. (2017)
Silver-rich TiN/Ag nano-multilayers	rBMSCs	Subcutaneous implantation in rats	Antibacterial activity and osteogenic differentiation	*In vitro* and *in vivo*		Wan et al. (2020)
AgNPs	mouse kidney-derived stem cells (mKSCs)		Enhanced podocyte differentiation	*In vitro*		Chowdhury et al. (2018)
AgNPs	mMSCs	Mouse model of bone fracture	Enhanced osteogenic differentiation	*In vivo*		Zhang et al. (2015)
TiO_2_/hydroxyapatite thin films	hMSCs/mouse mesenchymal tumor stem cell line ST-2		Enhanced osteogenic differentiation, adhesion and proliferation	*In vitro*		Jalali et al. (2020); Blendinger et al. (2021)
Ti- implants with zinc-modified calcium silicate coatings	rat bone marrow-derived pericytes	Ovariectomized rabbits	Promoted osteogenic differentiation	*In vivo*	TGF-beta/Smad signaling pathway	Yu et al. (2017)
TiO_2_-nanorods	murine macrophage cell line RAW264.7 and BMSCs	Femur marrow cavities of rabbits	Enhanced osteogenic differentiation and M1-to-M2 transition of macrophages	*In vitro* and *in vivo*		Yang et al. (2020)
TiO_2_ nanotubes	rBMSCs		Enhanced osteogenic differentiation	*In vitro*	Polymerization of F-actin enhanced the expression of RhoA and transcription factors YAP/TAZ	Tong et al. (2020); Liu et al. (2021)
Sr-loaded nanolayer on plasma sprayed Ca-Si coating	hBMSCs		Enhanced osteogenic differentiation and inhibited osteoclastogenesis	*In vitro*	Activated integrin 1and extracellular calcium sensitive receptor (CaSR)	Hu et al. (2017)
Sr-doped nanowire	RAW264.7 and rBMSCs		Improved osteogenic activities and reduced inflammatory reactions	*In vitro*	Enhancement of CaSR expression and further PKC and ERK1/2 phosphorylation	Li et al. (2018)
Fe_3_O_4_ NPs)/GO	rBMSCs		Intracellular ROS scavenging and osteogenic differentiation	*In vitro*		Zhang et al. (2020)
magnetic iron oxide NPs	hBMSCs		Enhanced osteogenic differentiation	*In vitro*	Upregulated long noncoding RNA INZEB2	Wang et al. (2017)
Fe_3_O_4_ NPs	hADSCs/Primary mice Osteoblast cells		Enhanced osteogenic differentiation	*In vitro*		Pistone et al. (2019); Kim et al. (2020b)
Fe_3_O_4_ NPs, chitosan and calcium-phosphate nanoflakes	hADSCs		Enhanced osteogenic differentiation	*In vitro*		Lee et al. (2019b)
Fe_3_O_4_ NPs	hUVECs	Skin injury	Promoted proliferation, migration, and angiogenesis	*In vitro* and *in vivo*		Li et al. (2020c)
MnO_2_ with ECM	hiPSC-NPCs	Mouse spinal cord injury	Enhanced neural differentiation	*In vivo*		Yang et al. (2018)
MnO_2_ nanocatalysts	hADSCs	Chick chorioallantoic membrane	Antioxidant and promoted angiogenesis	*In vitro*		Teo et al. (2019)
MnO_2_ NPs	hMSCs	Rat spinal cord injury	Antioxidant and neural differentiation	*In vivo*		Li et al. (2019)
Mesoporous silica NPs	hBMSCs		Enhanced osteogenic differentiation	*In vitro*		Tavares et al. (2021)
Silica-coated AuNPs	hMSCs		Enhanced osteogenic differentiation	*In vitro*		Gandhimathi et al. (2019)
BPNMs	rBMSCs		Enhanced neural differentiation	*In vitro*		Xu et al. (2020)
BPNMs	MC3T3-E1		Enhanced osteogenic differentiation	*In vitro*		Lee et al. (2019a)
BPNMs	Human dental pulp stem cells	rabbit model of bone defects	Enhanced osteogenic differentiation	*In vivo*	The bone morphogenic protein runt-related transcription factor 2 pathway	Huang et al. (2019)

Previous studies have suggested that the efficacy of stem cell transplantation depends on the differentiation into specific cell types, but there is growing evidence that the efficacy depends on paracrine behavior, which produces neuroprotective, angiogenesis, and immunomodulatory effects through the secretion of a large number of cytokines and proteins. MnO_2_ nanoparticles increase the secretion level of pro-angiogenic factors in MSCs (Teo et al., 2019). Li et al. (2020c) reported that exosomes produced by Fe_3_O_4_ NPs-treated hMSCs significantly promoted the angiogenesis of hUVECs. In addition, cell-derived nanoparticles have emerged as a promising alternative to synthetic nanocarriers for safer clinical outcomes (Chakravarti et al., 2020). Exosom-based therapies can effectively circumvent the toxicity of nanomaterials and the immune rejection problems associated with cell transplantation, with a broader application prospect in the field of regenerative medicine.

## Conclusion and Perspectives

Stem cell transplantation has enabled the cure of many diseases. Based on previous studies, we know that the unique physicochemical characteristics of inorganic NMs greatly influence stem cell fate (Shao et al., 2018; Hashemi et al., 2020), and the combination of inorganic NMs and stem cells provides new insights into the treatment of several diseases, such as bone injury and neurological disorders (Gandhimathi et al., 2019; Zhang et al., 2019). Inorganic NMs, as vehicles, can effectively deliver soluble factors such as growth factors and cytokines to induce stem cell differentiation, and can also interfere stem cell survival, homing and paracrine behaviors by forming specific patterns with fibrous/hydrogel scaffolds (Qi et al., 2017; Zhang et al., 2019).

Biosafety issues are one of the main reasons for the low clinical translation efficiency of inorganic NMs in the field of regenerative medicine, so we should also perform more comprehensive and systematic studies on the biosafety of inorganic NMs, which are not limited to superficial cytotoxicity tests, but should pay more attention to the *in vivo* distribution, visceral toxicity, as well as metabolic pathways of the NMs. Next, HSCs are known to play an important role in the field of regenerative medicine. But there are few publications related to inorganic NMs’ role in HSCs (England et al., 2013; Bari et al., 2015). Based on the advantages of inorganic NMs, it is significant to explore the effect of inorganic NMs on the fate of HSCs and the derived therapeutic effect. Furthermore, most of the researches on inorganic NMs focus on the differentiation of stem cells into terminal functional cells, such as bone cells or neurons (Shao et al., 2018; Tong et al., 2020). The limited efficacy is accompanied by safety problems. Therefore, it is more promising to turn the research hotspot to the exosomes secreted by stem cells stimulated by inorganic NMs. Exosomes are rich in active molecules while having lower toxicity and can be used for the treatment of more diseases.

In conclusion, inorganic NMs enrich the applications of stem cells, and there are still many problems to be solved, but nanomaterials combined with stem cell therapy is promising and will lead to major breakthroughs in the near future.
